# SOCS Proteins: Key Players in Immune Regulation During SARS‐CoV‐2 Infection

**DOI:** 10.1002/eji.202451645

**Published:** 2025-08-22

**Authors:** Juber Herrera‐Uribe, Nigel J. Stevenson

**Affiliations:** ^1^ Viral Immunology Group School of Biochemistry and Immunology Trinity Biomedical Sciences Institute Trinity College Dublin Dublin Ireland

**Keywords:** SOCS proteins, COVID‐19, cytokine signaling, viral infections

## Abstract

Suppressor of cytokine signaling (SOCS) proteins are crucial components of the immune response against viral infections. SOCS proteins inhibit cytokine signaling through various mechanisms, such as blocking STAT binding to JAKs and targeting proteins for ubiquitination and degradation. While these proteins maintain immune balance by suppressing excessive inflammatory responses, many viruses, including SARS‐CoV‐2, exploit SOCS proteins to evade host immunity. In consequence, understanding their modulatory functions in viral disease has become increasingly relevant. Therefore, this review aims to describe and discuss studies involving SOCS expression data in COVID‐19 and their potential modulation as a valuable use for therapeutic strategies.

## Graphical abstract

SOCS proteins are key modulators of cytokine signaling during SARS‐CoV‐2 infection, balancing antiviral responses and inflammation. The virus differentially regulates SOCS expression across tissues and disease stages, exploiting SOCS regulatory function to suppress antiviral pathways. This regulatory role positions SOCS proteins as potential targets for stage‐specific immunomodulatory therapies.

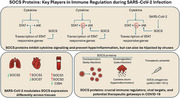

## 1 Introduction

SOCS proteins constitute a family of eight intracellular proteins with diverse regulatory roles [1]. These proteins exhibit a multifaceted regulatory function by influencing various signaling pathways, including the Janus kinase‐signal transducer and activator of transcription (JAK/STAT), nuclear factor kappa B (NF‐κB), mitogen‐activated protein kinase (MAPK), and receptor tyrosine kinase (RTK) pathways [2, 3, 4, 5]. SOCS proteins employ several methods to inhibit cytokine signaling, including the binding of JAKs and receptor chains to block STAT binding sites, and the targeting of receptor complex components for ubiquitination and subsequent proteasomal degradation [6, 7].

Each SOCS protein consists of three distinct domains: an N‐terminal domain, a central Src homology 2 (SH2) domain, and a highly conserved C‐terminal SOCS box [8]. The N‐terminal domain, varying in length, plays a role in modulating SOCS functions. SOCS1‐3 and CIS contain a relatively short N‐terminal (50–80 residues), while SOCS4‐7 have elongated N‐terminals, extending up to 380 residues [9] Notably, SOCS1 and SOCS3 possess a kinase inhibitory region (KIR) within their N‐terminal domain, which enables them to inhibit JAK activity [7, 10]. SOCS4 and SOCS5 incorporate an N‐terminal conserved region (NTCR), and although the function of NTCR remains largely unknown, it has been implicated in the inhibition of JAK1 [11]. The central SH2 domain plays a crucial role in interacting with phosphotyrosine (pY) residues on target proteins, including cell surface receptors and JAKs [12]. In all SOCS proteins, an extended SH2 sequence (ESS) adjacent to the SH2 domain contributes to high‐affinity binding of the SH2 domain to the target pY [13]. For instance, the SH2 domain of SOCS1 binds to the activation loop of JAK2, while the SH2 domains of SOCS2 and SOCS3 interact with pY residues on the growth hormone and gp130 receptors, respectively [7, 14, 15]. The SOCS box, a C‐terminal motif found in all SOCS proteins, is essential for recruiting the ubiquitin‐transferase system and is implicated in protein stability [16]. The SOCS box consists of two subdomains: the BC box region, responsible for recruiting Elongin B and C, and the Cul box, which mediates Cullin 2 and Cullin 5 binding. This complex formation, including Elongin B, Elongin C, Cullin 2 or Cullin 5, and RING box protein 2 (RBX2), is critical for the ubiquitination process [16].

SOCS proteins play a crucial role in maintaining immune balance by providing negative feedback regulation of cytokine signaling, thereby preventing excessive immune responses that could harm the host during infection [17]. The activation of the NF‐κB pathway, a central mediator of inflammation, can occur through mechanisms that bypass SOCS control. While SOCS1‐mediated ubiquitination and degradation of the NF‐κB p65 subunit limit NF‐κB‐driven transcription [18, 19], other studies indicate that its regulatory influence is limited. Prele et al. 2008 demonstrated that SOCS1 does not significantly affect the early activation or DNA‐binding capacity of NF‐κB following TLR4 stimulation, suggesting that initial NF‐κB‐mediated inflammatory responses proceed without SOCS1 intervention [20]. However, Jinxia et al. 2020 [21] showed that SOCS1 overexpression attenuated the expression of TLR4 in BV2 cells, while the knockdown of SOCS1 upregulated TLR4.

SOCS regulation is especially important when viruses invade the host. Viruses are recognized by host immune cells through pathogen‐associated molecular patterns (PAMPs) [22]. Host immune and epithelial cells have innate immune receptors that recognize, discriminate, and react to viral infections [23]. Innate immunity is the earliest immune mechanism to be activated once the virus invades the host. The host cells identify PAMPs in nucleic acids or viral proteins through PRRs, including toll‐like receptors (TLRs) and others, such as RIG‐I‐like receptor (RLR) and nucleosides acid‐binding oligomeric domain (NOD)‐like receptor (NLR). This promotes host cells to generate the antiviral, type I interferon (IFN‐I) response. Proinflammatory cytokine production also mounts an effective regulation of the immune response and ensures normal activities of the host cells [24].

While the immune response is vital for viral clearance, excessive activation can lead to immune dysregulation. Dysregulated cytokine activity during viral infections can cause tissue damage and organ dysfunction [25]. Notably, viruses have developed strategies to manipulate host immune responses through modulation of SOCS protein expression, blocking pathways, such as the JAK/STAT signaling cascade. This manipulation reduces antiviral responses and dampens the innate and adaptive immune responses, favoring viral replication [26, 27]. Studies have shown how viruses can modulate SOCS proteins to evade the immune system, thus inhibiting the expression of IFN‐stimulated genes (ISGs) and the IFN type I response. Hepatitis B and C virus (HBV and HCV), herpes simplex virus 1 (HSV‐1), influenza viruses, respiratory syncytial virus (RSV), zika virus (ZIKV), human immunodeficiency virus (HIV), Semliki forest virus (SFV), coxsackievirus, Ebola virus, varicella zoster virus (VZV), west Nile virus (WNV), porcine reproductive and respiratory syndrome virus (PRRSV), Japanese encephalitis virus (JEV), and Epstein–Barr virus (EBV) have all been observed to modulate SOCS proteins for their own benefit to avoid the innate immune response [28, 29, 30, 31, 32, 33, 34, 35, 36, 37, 38, 39, 40, 41]. Huang et al. summarized a list of viruses that hijack mainly SOCS1 and/or SOCS3 to interfere with interferon (IFN) signaling and proinflammatory responses, thereby enhancing viral replication. For instance, PRRSV upregulates SOCS1, leading to reduced IFN‐β and ISG expression, which promotes viral replication. HCV induces SOCS3, suppressing IFN‐α/β and NF‐κB activation, and dampening inflammatory responses, facilitating replication. Similarly, HSV‐1 also induces SOCS3 expression, which inhibits IFN‐α/β signaling. Influenza A virus (IAV) upregulates both SOCS1 and SOCS3, reducing type I and II IFNs while increasing IFN‐λ, thereby facilitating viral persistence. RSV increases SOCS1 and SOCS3 expression, which diminishes IFN signaling, chemokine production, and ISG expression. WNV elevates SOCS1 levels, suppressing type I IFN signaling, while ZIKV targets both SOCS1 and SOCS3 to inhibit type I and III IFN responses, promoting viral replication. These examples collectively demonstrate how viruses fine‐tune host immune signaling via SOCS proteins to escape detection and create a favorable environment for replication and persistence [24].

Additionally, growing evidence demonstrates that viruses have evolved sophisticated mechanisms to modulate SOCS proteins, particularly SOCS1 and SOCS3, as a means of evading host innate immune responses. These strategies often involve hijacking host signaling pathways or inducing transcriptional changes that modulate SOCS expression, ultimately impairing interferon signaling and inflammatory responses. For example, HCV uses its p7 protein to upregulate SOCS3 via STAT3 and ERK‐mediated signaling, which suppresses the host inflammatory response to TNF‐α, thus dampening antiviral immunity [42]. RSV modulates SOCS activity through its nonstructural protein 1 (NS1), which induces SOCS1 and SOCS3 expression to block type I interferon (IFN) responses and chemokine production, contributing to viral persistence [30]. In the case of West Nile virus (WNV) and tick‐borne encephalitis virus, infection results in transcriptional upregulation of SOCS1 and SOCS3, which suppress antiviral cytokine responses [43]. Japanese encephalitis virus (JEV) also modulates SOCS expression: infected macrophages show increased SOCS1 and SOCS3 mRNA, which correlates with reduced IFN signaling and impaired innate immune responses, facilitating viral replication [38]. In the context of EBV, the virus upregulates SOCS1 and SOCS3 during latent and lytic phases of infection, likely through viral protein‐induced activation of host STAT3, which enhances SOCS transcription and inhibits downstream antiviral signaling [44]. Collectively, these studies demonstrate that SOCS protein manipulation is a common viral strategy, often achieved via direct activation of host signaling cascades (e.g., JAK/STAT, ERK) or viral protein interaction with host immune modulators. This fine‐tuned regulation allows viruses to suppress type I and II IFN pathways, dampen proinflammatory signaling, and persist within the host despite active immune surveillance.

## 2 Immune Regulation in SARS‐CoV‐2 Infection by SOCS Proteins

Key cytokines that prevent viral replication are type I IFNs, such as IFN‐α and β. Other cytokines, such as IL‐8, TNF‐α, IL‐1, and IL‐6, activate immune cells and trigger an inflammatory response [45]. However, inappropriate activation of the immune response can lead to dysregulation of host cytokine activity during infection and cause tissue damage and organ dysfunction. Notably, previous studies on SARS‐CoV reported the overexpression of SOCS3 in B lymphoma cells, reducing the antiviral effect of IFN [46], also SOCS3 upregulation was observed by Okabayashi et al. 2006 [47] in an in vitro SARS‐CoV infection of the human colon carcinoma cell line, Caco2, and the alveolar epithelial cell line, A549. Interestingly, the induction of SOCS3 by SARS‐CoV was significantly lower compared with RSV infection in the same cell lines. Given the genome similarity between SARS‐CoV and SARS‐CoV‐2, both viruses may modulate SOCS proteins using similar mechanisms to evade the immune response. However, studies focusing on SARS‐CoV‐2 evasion of the innate immune system through SOCS protein modulation are still limited, although the dysregulation of the JAK/STAT pathway has been reported during COVID‐19 disease, and this pathway is tightly linked with SOCS protein activity [48].

Initial in silico studies suggested that SARS‐CoV‐2 could be using its viral microRNAs to target host genes. Aydemir et al. 2021 [49] predicted 40 SARS‐CoV‐2 microRNA interactions with host genes, including all members of the SOCS family, except CISH, suggesting that the expression of the viral microRNAs modulate the immune system through SOCS.

**FIGURE 1:**
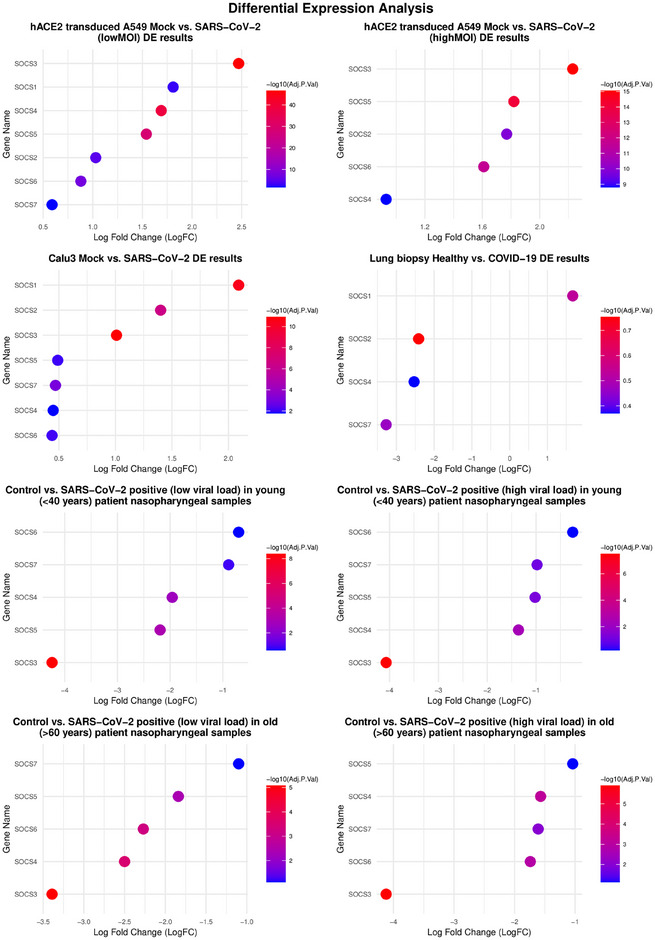
SOCS gene log fold change values from the study by Singh et al. 2021 [50]. RNA‐seq data sets from different studies were obtained from in vitro infections with SARS‐CoV‐2 and IAV. Also, comparisons of nasopharyngeal and lung biopsy samples from healthy donors and COVID patients were performed.

Singh et al. 2021 [50] performed a comparative gene expression analysis, extracting RNA sequencing data from different studies that included SARS‐CoV‐2 in vitro infection on two lung carcinoma cells lines, hACE2 transduced A549 (expressing human ACE2 receptor) and Calu3 [50], the A549 infection model included two multiplicity of infection (MOI, high = 2 and low = 0.2). Additionally, the analysis included data sets from lung biopsy and nasopharyngeal samples that were segregated by age and viral load. In Figure 1, we extracted the differential gene expression values of SOCS genes from multiple comparisons performed by Singh et al. 2021 [50]. Interestingly, the differential expression of SOCS genes changed according to the biological model used for the study. In the hACE2‐transduced A549 cell line, SOCS genes were overexpressed even using low (low MOI, SOCS1‐7) or high MOI (high MOI, SOCS2‐6). A similar tendency was observed in Calu3 cells, where SOCS1‐7 were upregulated. However, SOCS genes (SOCS3‐7) were downregulated in nasopharyngeal samples from COVID‐19 patients across age groups and viral load, compared with healthy donors. A similar tendency was observed in lung biopsies from COVID‐19 donors (compared with healthy donors), where only the SOCS1 gene was upregulated and SOCS2/4/7 downregulated. Disease course or disease severity scores from COVID‐19 donors were not reported. Taken together, the differences in SOCS gene expression observed might reflect a tissue‐specific response in which local immune suppression is altered compared with the in vitro cell models.

Infection with SARS‐CoV‐2 can manifest as asymptomatic or exhibit varying degrees of severity. Severe cases of COVID‐19, which can lead to fatality, are characterized by a pulmonary and multiorgan disease involving different tissues such as the heart, vascular system, kidneys, lungs, and other organs and tissues [51]. Autopsies of individuals who have died of COVID‐19 reveal notable pathological features in the lungs, including diffuse alveolar damage, severe endothelial injury associated with intracellular virus, and disrupted cell membranes [52]. Pulmonary vessels display widespread thrombosis and microangiopathy. The inflammatory condition and thrombosis extend to varying degrees into the heart, gut, and kidneys [53], which means that SOCS expression must also be evaluated in different tissues from COVID‐19 patients to understand its impact on the regulation of the antiviral response.

**FIGURE 2:**
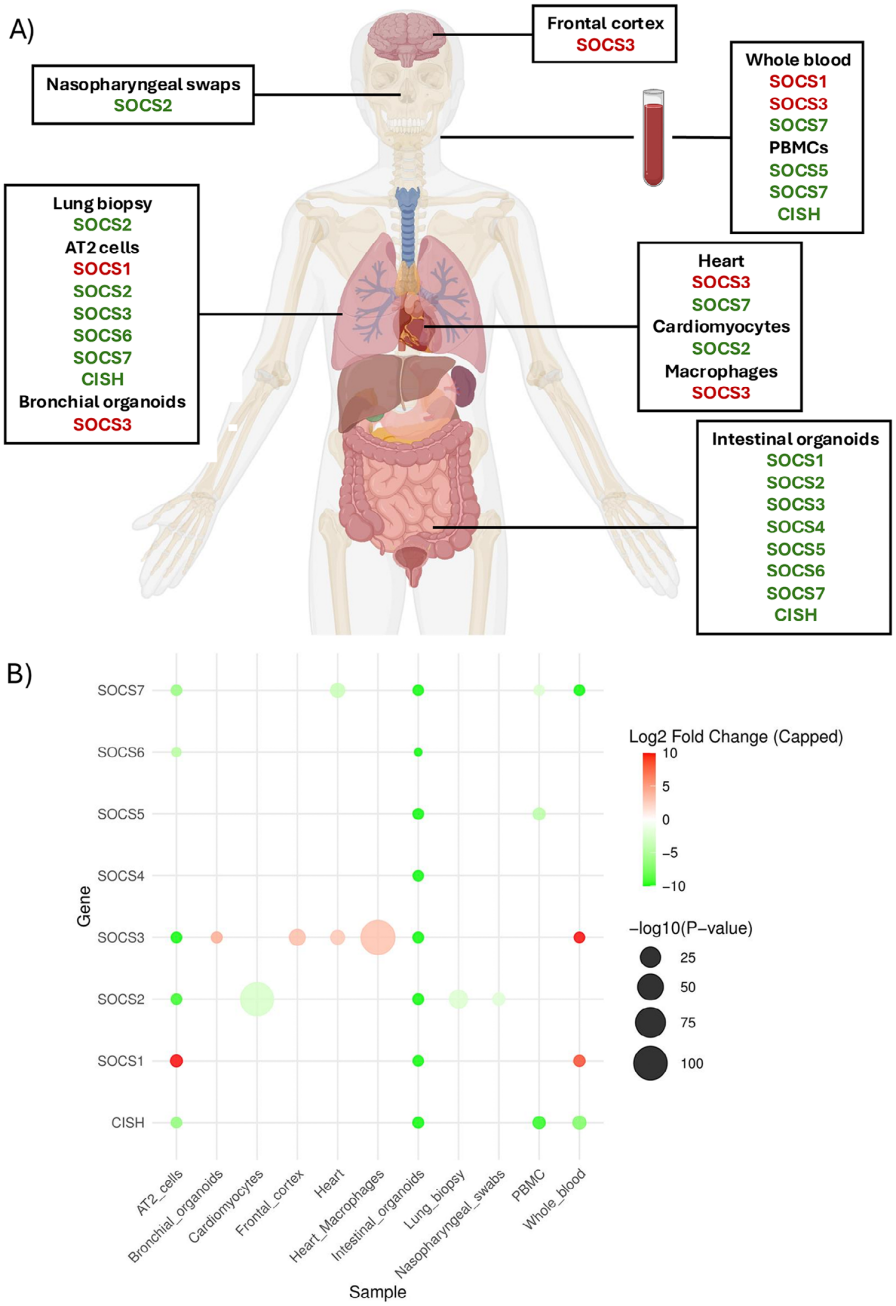
Tissue‐specific expression patterns of SOCS genes in response to SARS‐CoV‐2 infection. (A) Statistically significant, differentially expressed SOCS genes were extracted from SCovid v2.0 database and represented as overexpressed (red) and downregulated (green) in different tissues/cells samples from COVID‐19 donors compared with healthy donors. (B) Dot plot showing Log2 fold change values of differentially expressed SOCS genes in tissues/cells as shown in (A). Each dot represents a gene‐sample pair, with the x‐axis indicating the sample type and the y‐axis listing the genes. The color of the dots corresponds to the Log2 fold change in gene expression, where red indicates upregulation (positive Log2 fold change), green indicates downregulation (negative Log2 fold change), and white represents values close to zero (no significant change). The size of the dots reflects the statistical significance of the expression change, scaled by the −log10(*p*‐value). Larger dots denote more significant changes (lower *p*‐values). Extreme log2 fold change values were capped at −10 and 10 to ensure a balanced and interpretable color gradient. AT2 cells, alveolar type 2 cells; PBMC, peripheral blood mononuclear cells [55].

As mentioned before, studies focusing on the role of SOCS proteins in SARS‐CoV‐2 infection are still limited. However, the evaluation of whole transcriptomes from different tissues and isolated primary cells has been extensively studied using gene expression technologies, such as bulk RNA sequencing and single‐cell RNA sequencing data [54]. We used available data from SCovid v2.0, a gene expression atlas that integrates data from SARS‐CoV‐2 and other human coronaviruses infections, to extract SOCS gene expression from different tissues and cells that were obtained either by bulk or single‐cell RNA sequencing [55]. As shown in Figure 2, using the SCovid v2.0 database, we summarized all tissues and resident cells where SOCS genes were differentially expressed from patients with COVID‐19, compared with healthy donors (Figure 2). We found SOCS genes differentially expressed in tissues and resident cells from the brain, nasopharyngeal cavity, lungs, heart, intestine, and blood cells. Interestingly, all SOCS genes within the SOCS family do not have the same expression pattern in samples from COVID‐19 patients. SOCS1 and SOCS3 were upregulated in specific tissues, including the frontal cortex, AT2 cells, bronchial organoids, whole blood, resident macrophages in the heart and the heart tissue, whereas other SOCS family members (SOCS2, SOCS4‐7, and CISH) tended to be downregulated in response to SARS‐CoV‐2 infection (Figure 2A).

A more detailed representation of SOCS expression changes across tissues is provided in the dot plot (Figure 2B), which quantifies fold change values and statistical significance. SOCS3 upregulation was particularly high in whole blood and low *p*‐values in resident macrophages in the heart, supporting its role in modulating inflammation in COVID‐19. SOCS1 was predominantly upregulated in the lungs (AT2 cells), indicating a potential contribution to immune dysregulation in the pulmonary compartment. In contrast, SOCS3, SOCS7, and CISH were significantly downregulated in intestinal organoids, SOCS2‐3, SOCS6‐7, and CISH in AT2 cells, SOCS2 in nasopharyngeal swaps and lung samples, SOCS7 in heart, and SOCS5, SOCS7, and CISH in whole blood and peripheral blood mononuclear cells (PBMCs). This tissue‐specific modulation of SOCS expression adds a layer of complexity to our understanding of the host‐SARS‐CoV‐2 interaction and the potential modulation of SOCS genes in antiviral immunity. Taken together, SARS‐CoV‐2 can modulate SOCS gene expression in different ways according to cell/tissue or biological model, and that could be crucial for the understanding of the infection, as well as the development of therapeutic for COVID‐19.

## 3 SOCS1/3 Dysregulation in COVID‐19: Pathogenic Mechanisms, Comorbidities, and Therapeutic Implications

Understanding how SARS‐CoV‐2 strategically manipulates the host immune system through SOCS proteins has potential clinical implications for managing COVID‐19 and possibly other CoVs. This knowledge opens alternatives for the development of targeted therapies aimed at modulating SOCS proteins, with the aim of restoring a more effective antiviral response. By unraveling the complex balance of SOCS proteins in the immune system, immunomodulatory treatments could be designed to regulate SOCS levels, potentially enhancing the host's ability to induce an anti‐viral state against SARS‐CoV‐2, without inducing excessive inflammation.

SOCS1 and SOCS3 are increasingly recognized as central regulators of host immune responses during viral infections [56, 57, 58]. These proteins are well known to inhibit the JAK/STAT pathway [59], dampening type I and II interferon signaling and impairing early antiviral immunity. SOCS1 and SOCS3 are typically induced by coronaviruses in early‐stage infection. SOCS1 and SOCS3 induction interfere with the normal JAK2 and TYK2 activity by binding the functional domain of 12 amino acids called the kinase inhibitory region (KIR), which inhibits STAT activation and subsequently delays IFN type I and II production, thus affecting the antiviral response at an early stage [60, 61].

Different studies have suggested small peptide antagonists of SOCS1 and SOCS3 [62]. The antagonist, pJAK2 (1001‐1013), is a SOCS1 and SOCS3 inhibitor that binds to the KIR region, blocking their inhibitory activity in different viral infections, such as HSV‐1 and influenza A [63, 64]. Frey et al. 2009 [63] demonstrated the double antiviral effect that pJAK2 (1001‐1013) had by inhibiting the SOCS1 and SOCS3 activity in keratinocytes infected with HSV‐1, as well as a synergistic effect when combined with IFNγ treatment. A similar antiviral effect was proved by Ahmed et al. 2015 [64] in mice infected with the influenza A virus, where the use of pJAK2 (1001‐1013) prevented the typical weight loss and drop in body temperature and induced the cellular and humoral response to the influenza virus. Ahmed et al. 2015 [64] also tested the pJAK2 (1001‐1013) in Calu3 cells in SARS‐CoV‐2 infection. Pretreatment of pJAK2 (1001‐1013) in different concentrations (3, 10, and 30 µM) significantly reduced the replication and release of SARS‐CoV‐2, suggesting the potential use of pJAK2 (1001‐1013) as an antiviral drug for the treatment of SARS‐CoV‐2 infection [65]. However, the use of SOCS inhibitors in late stages of SARS‐CoV‐2 infection may enhance the cytokine storm, the principal reason for the acute respiratory distress syndrome (ARDS). Mahmudpour et al. 2020 [66] highlighted how uncontrolled proinflammatory signaling contributes to ARDS, the leading cause of mortality in severe COVID‐19 cases. Thus, the use of SOCS inhibitors at different COVID‐19 stages must be evaluated. A review by Low et al. 2021 [58] further emphasized this dual role of SOCS proteins, describing them as both protective and pathogenic depending on the disease stage and immune environment.

Importantly, SOCS regulation appears to change not only by infection stage but also by host genetic and epigenetic factors. A study by Dobrindt et al. 2023 [67] found that promoter hypomethylation of the SOCS1 gene was significantly associated with severe COVID‐19 outcomes, while a SOCS1 polymorphism (rs33989964) was linked to increased disease severity. These findings suggest that individuals with altered SOCS1 epigenetic profiles may exhibit impaired interferon responses and more severe disease progression. Additionally, miRNAs have emerged as key posttranscriptional regulators of SOCS1 expression. For example, miR‐155, known to be upregulated in severe viral infections, targets SOCS1 and promotes inflammatory signaling [68]. Bioinformatic and in vitro analyses suggest that high miR‐155 activity during SARS‐CoV‐2 infection may suppress SOCS1, contributing to the excessive inflammation observed in severe cases [69]. Clinical studies have shown that during SARS‐CoV‐2 infection, miR‐155 expression increases progressively with disease severity, while SOCS1 expression declines. This inverse relationship correlates with a skewed Th17/Treg balance and elevated inflammation, contributing to poor outcomes [70, 71, 72]. A similar mechanism has been described in HIV‐1 infection, where SOCS3 is downregulated early during infection, resulting in enhanced NF‐κB activation and proinflammatory cytokine production. This inflammatory state creates a favorable environment for viral replication and persistence and also sets the stage for chronic immune activation [73]. More broadly, SOCS1 itself functions as a critical checkpoint of immune homeostasis, as highlighted by Hixon et al. 2024 [74]. Its loss or suppression removes a key brake on cytokine signaling, particularly the JAK/STAT and NF‐κB pathways, leading to exaggerated inflammation and increased susceptibility to autoimmune and chronic inflammatory conditions. Together, these findings demonstrate that while many viruses upregulate SOCS proteins to escape early immune detection, others (or the host response to them) can cause SOCS1 and SOCS3 downregulation, which exacerbates hyperinflammation and immune imbalance, a hallmark of severe and chronic viral disease.

Notably, SOCS1 and SOCS3 dysregulation intersect with host comorbidities such as obesity, type 2 diabetes, and metabolic syndrome, all of which are strongly associated with increased COVID‐19 severity and mortality [75, 76]. In these conditions, chronic low‐grade inflammation and elevated levels of circulating leptin and pro‐inflammatory cytokines lead to persistent activation of the JAK/STAT pathway, resulting in upregulation of SOCS1 and SOCS3 in immune and metabolic tissues [77, 78, 79]. This elevated SOCS1 and SOCS3 expression contributes to leptin and insulin resistance in immune cells, impairing their ability to respond to infection effectively.

Wunderlich et al. 2013 [80] and Carow & Rottenberg et al. 2014 [81] previously demonstrated that SOCS3‐mediated feedback inhibition of leptin receptor (Ob‐Rb) signaling in macrophages and other immune cells results in impaired cytokine production and immune cell metabolism, thereby compromising early antiviral responses. Extending this understanding, Muskiet et al. 2022 [82] proposed a detailed mechanistic model in which SOCS1 and SOCS3 act as central molecular links between obesity‐induced leptin resistance and impaired type I interferon (IFN‐I) responses during SARS‐CoV‐2 infection. Specifically, under normal conditions, leptin enhances the IFN‐I response through JAK2/STAT1‐mediated transcription of interferon‐stimulated genes (ISGs). However, in obesity, chronically elevated leptin levels induce SOCS1 and SOCS3, which in turn inhibit JAK2 phosphorylation and downstream STAT1/STAT2 activation. This inhibition leads to suppression of ISG expression and reduced antiviral defense, thereby compromising the early innate immune response crucial for controlling SARS‐CoV‐2 replication. This suppression of leptin and IFN signaling creates an immunometabolic environment in which viral clearance is delayed and proinflammatory cytokine production is amplified at later stages, contributing to the development of cytokine storms and severe disease progression. Therefore, SOCS‐mediated leptin and interferon resistance offers a compelling explanation for the increased vulnerability of obese individuals to severe COVID‐19 [82].

SOCS1 and SOCS3 regulatory function in antiviral response highlights the need for personalized medicine approaches, considering the diverse expression patterns of SOCS proteins across different tissues and individuals. Integrating this knowledge into combination therapies alongside existing antiviral drugs or immunomodulators may offer a more comprehensive strategy [83]. Additionally, understanding how SOCS proteins influence vaccine efficacy can inform the optimization of vaccine development, especially in individuals with comorbidities, who induce poor antibody levels after vaccination [84, 85]. The differential expression of SOCS proteins in various tissues may also serve as biomarkers for predicting disease severity and guiding clinicians in selecting treatment strategies more effectively.

## 4 Conclusion and Future Direction

Despite some progress in understanding the interaction between SARS‐CoV‐2 and SOCS proteins, much remains unclear about how this virus regulates SOCS proteins and its effects on the immune response over the course of the disease. Key areas for further investigation include the precise mechanisms by which SARS‐CoV‐2 manipulates SOCS proteins and their impact on cytokine signaling, given the critical roles of cytokines in immune regulation and COVID‐19 pathogenesis, particularly in severe cases with cytokine storms.

The timing and level of SOCS protein expression across different tissues during SARS‐CoV‐2 infection also require attention. Host factors, such as comorbidities that influence SOCS expression and immune responses, should be explored, as they may affect disease outcomes. Furthermore, understanding the role of SOCS proteins in long COVID could provide insights into persistent postinfection symptoms. Comparative studies with other coronaviruses and targeted SOCS therapies hold promise for advancing therapeutic options in COVID‐19, especially those aimed at mitigating cytokine storms without compromising the antiviral response.

## Author Contributions

All authors contributed to the review conception and design. Figures elaboration was performed by Juber Herrera‐Uribe. The first draft of the manuscript was written by Juber Herrera‐Uribe, and Nigel J. Stevenson commented and edited on previous versions of the manuscript. Supervision and grant obtaining were performed by Nigel J. Stevenson. All authors read and approved the final manuscript.

## Conflicts of Interest

The authors declare no conflicts of interest.

## Peer Review

The peer review history for this article is available at https://publons.com/publon/10.1002/eji.202451645.

## Data Availability

The data that support the findings of this study are available in Singh et al., 2021 and Qi et al., 2022 at https://doi.org/10.3389/fgene.2021.599261 and https://doi.org/10.1093/nar/gkab881, respectively.
